# Relationship between Vascular Calcification, Protein-Energy Wasting Syndrome, and Sarcopenia in Maintenance Automated Peritoneal Dialysis

**DOI:** 10.3390/life11070666

**Published:** 2021-07-07

**Authors:** Gustavo Leal-Alegre, Claudia Lerma, Gabriela Leal-Escobar, Bernardo Moguel-González, Karen Belén Martínez-Vázquez, Karla Berenice Cano-Escobar

**Affiliations:** 1 Department of Nephrology, Instituto Nacional de Cardiología Ignacio Chávez, Mexico 04480, Mexico; gztvo@comunidad.unam.mx (G.L.-A.); Gabriela.leal@cardiologia.org.mx (G.L.-E.); bernardo.moguel@cardiologia.org.mx (B.M.-G.); kb_sery@comunidad.unam.mx (K.B.M.-V.); 2 Department of Electromechanical Instrumentation, Instituto Nacional de Cardiología Ignacio Chávez, Mexico 04480, Mexico or dr.claudialerma@gmail.com (C.L.)

**Keywords:** end-stage renal failure, dialysis, malnutrition, sarcopenia, vascular calcification

## Abstract

Vascular calcifications affect 80% to 90% of chronic kidney disease patients and are a predictive factor of cardiovascular mortality. Sarcopenia and protein-energy wasting syndrome are also associated with mortality. The aim was to assess the relationship between vascular calcification, sarcopenia, and protein-energy wasting syndrome (PEW) in automated peritoneal dialysis patients. Fifty-one maintenance automated peritoneal dialysis patients were included (27 were male, mean age 39 ± 14 years). Vascular calcification was assessed based on abdomen, pelvis, and hand radiographs. Sarcopenia was assessed with bioimpedance analysis and a hand grip strength test. The Malnutrition–Inflammation Score and the presence of PEW were also assessed. Vascular calcification was present in 21 patients (41.2%). Univariate logistic regression analysis showed that age (*p* = 0.001), Malnutrition–Inflammation Score (*p* = 0.022), PEW (*p* = 0.049), sarcopenia (*p* = 0.048), and diabetes (*p* = 0.010) were associated with vascular calcification. Multivariate logistic regression analysis showed that age (*p* = 0.006) was the only variable associated independently with vascular calcification. In conclusion, there is association between vascular calcification, PEW, and sarcopenia in patients with maintenance automated peritoneal dialysis. These associations are not independent of age. This demonstrates the importance of nutritional status in the prevention of vascular calcification.

## 1. Introduction

Chronic kidney disease (CKD) is an important risk factor for cardiovascular disease. Patients with CKD have a 20-fold risk for cardiac-associated deaths [1]. In Mexico, cardiovascular-disease-associated deaths account for 51.6% of total deaths in peritoneal dialysis CKD patients [2]. Overall, 80% to 90% of CKD patients demonstrate the presence of vascular calcifications, which is a predictive factor of cardiovascular mortality [3]. Several risk factors for vascular calcification have been described. They are classified as traditional and non-traditional. The traditional factors are diabetes, dyslipidemia, age, genetics, and smoking. The non-traditional risk factors are inflammation, oxidative stress, advanced glycated products, bone mineral disease, and fibroblast growth factor 23 [4]. A study in 154 Chinese patients with CKD in peritoneal dialysis found that 64.9% have calcification in large vessels, 42.9% in medium size vessels, and 9.7% in small vessels. The factors they found associated with them were age, dialysis vintage, lower levels of parathormone, and Charlson comorbidity index [5]. A study evaluating the presence of calcification in hemodialysis patients with pelvis and hand radiographies found that vascular calcification is associated with coronary heart disease (*p* = 0.008), peripheral artery disease (*p* < 0.001), and vascular disease (*p* = 0.001) [6].

Sarcopenia is defined as the loss of skeleton muscle and strength associated with aging, disability, and chronic diseases. It has been associated with cardiovascular risk odds ratio (OR) = 1.768 (CI 1.075–2.909, *p* = 0.025) [7]. In CKD patients treated with peritoneal dialysis, the prevalence of sarcopenia has been reported in 8.4%, which was associated with higher levels of interleukin-6, hypoalbuminemia, higher mortality risk, and worse prognosis [8]. Protein-energy wasting syndrome (PEW) is a condition related to malnutrition and inflammation in CKD patients. Its diagnosis is made with a nutritional evaluation including physical exam, muscle mass, biochemical studies, and protein intake [9]. A phenomenon called “inverse epidemiology” has been reported in CKD, in which a higher body mass index has been associated with better survival, this is partially explained by the presence of PEW, which has been associated with higher mortality rates in CKD [10].

As described above, there is evidence of the association between vascular calcification, inflammation, and mineral bone disease with cardiovascular mortality in CKD. There has also been described association between sarcopenia and cardiovascular mortality. We found no studies comparing the presence of vascular calcification in automated peritoneal dialysis with the presence of sarcopenia and PEW. This study aims to evaluate the association between presence of vascular calcification, sarcopenia, and PEW in automated peritoneal dialysis patients.

## 2. Materials and Methods

### 2.1. Study Design and Patients

This is a single-center cross-sectional observational study, which enrolls patients with CKD who are on maintenance automated peritoneal dialysis in the Instituto Nacional de Cardiología Ignacio Chávez from January 2019 to April 2019. Inclusion criteria we used were age >18 years, automated peritoneal dialysis treatment, and stable clinical condition without hospitalizations for the last 3 months. Exclusion criteria were patients with pregnancy, active infection, trauma or surgery 30 days prior to the study, and amputations. All the subjects were treated with automated peritoneal dialysis, with a daily dialysate exchange dose of more than 6 L with glucose-based, lactate-buffered peritoneal dialysis solutions (Baxter: 1.5 and 2.5% solutions, sodium 132 mEq/L, calcium 3.5 mEq/L, magnesium 0.5 mEq/L, chloride 96 mEq/L, and lactate 40 mEq/L). The volume of the dialysate depended initially on the body surface area (calculated with Mosteller’s formula based on weight and height) and the presence or absence of residual urinary volume (to measure creatinine clearance). The adequacy of doses in the follow-up of the patient was modified by changes in the previously mentioned parameters or by inadequate clearance of solutes (evidenced with laboratory tests), symptoms of uremia, and the patient’s volume status.

We obtained demographic information and clinical characteristics for all patients. Data included age, gender, CKD duration, peritoneal dialysis duration, primary renal disease, the presence or absence of diabetes, hypertension, vital signs, body mass index, prior kidney transplant, and prior parathyroidectomy. Then, we calculated the Charlson comorbidity index of each patient. All participants gave their informed consent prior to their inclusion in the study. The study protocol was approved by the Research and Ethics Committees of our institution (protocol number: PT-19-114).

### 2.2. Laboratory Tests

Laboratory variables were assessed the same day as the radiographs in each patient. Laboratory results include complete blood count, serum corrected calcium, phosphorus, serum intact parathyroid hormone, alkaline phosphatase, albumin, total cholesterol, triglycerides, serum creatinine, blood urea nitrogen, hemoglobin, C reactive protein, and estimated residual renal function with 24 h urine creatinine clearance.

### 2.3. Vascular Calcification Assessment

**Figure 1:**
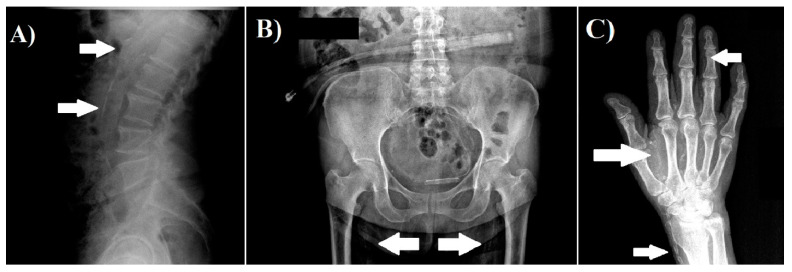
Examples of vascular calcification. (**A**) Lateral abdomen radiograph, which shows the presence of vascular calcification in aorta. (**B**) Pelvis radiograph, which shows the presence of vascular calcification in the iliac arteries. (**C**) Hand radiograph, which shows vascular calcification in the radial, palmar arch, and digital arteries.

We obtained radiographs of both hands, pelvis, and lateral abdomen to assess the presence of vascular calcification as described previously [5,11]. The lateral abdominal radiographs were divided into two sections by a horizontal line over the intervertebral space between L2 and L3; radiographs of the pelvis were divided by two lines: a horizontal line just above the femoral heads and a median vertical line; and radiographs of each hand were divided by a horizontal line over the proximal end of the metacarpals. The presence of calcification in any section was given 1 point, and scores from all sectors were summed up to a total score. The highest total score was 10 points. The radiographs were reviewed by a nephrologist. Examples of radiographs with vascular calcifications are shown in Figure 1.

### 2.4. Nutritional Assessment

Every patient was weighted, measured, and evaluated with bioelectrical impedance analysis (InBody S10, Seoul, Korea) in fasting status without fluid in the peritoneal cavity. We calculated the Malnutrition–Inflammation Score [12] in each patient, and we assessed the presence of PEW in patients with a total Malnutrition–Inflammation Score >8. and obtained the hand grip strength in both hands with hand grip dynamometer (JAMAR) three times, according to the Southampton protocol [13], to evaluate the presence of sarcopenia.

### 2.5. Statistical Analysis

In all continuous or ordinal variables, Kolmogorov–Smirnov tests were applied to assess for normal distribution. Variables with normal distribution are described with mean ± standard deviation and compared between groups with or without vascular calcification with t-Student test for independent samples. Variables without normal distribution were described as median and compared with Mann–Whitney U test. Logistic regression analyses were performed to assess the association between vascular calcification (as dependent variable) and variables with significant differences between patients with or without vascular calcification as independent variables. Nominal variables are described as absolute value and percentage, and they were compared between groups by chi-squared test or Fisher’s exact test. The SPSS (version 21.0, International Business Machines Corp., Armonk, NY, USA) statistical software was used for statistical analysis.

## 3. Results

It was found that 21 out of 51 patients had the presence of vascular calcification in at least one vessel, which corresponds to an estimated prevalence of vascular calcification of 41% (95% confidence interval 29–53%). Table 1 shows the distribution of calcifications. Vascular calcification was found in the large vessels of 15 patients (29%), in the medium vessels of 17 patients (33%), and in the small vessels of 13 patients (25%).

The sociodemographic variables and clinical characteristics are shown in Table 2. Compared to those without vascular calcification, patients with the presence of vascular calcification were older, had lower dialysis volume, were more likely to have diabetes, and had higher Charlson comorbidity index and lower mean values of diastolic and median blood pressure. There were no differences between groups in all other variables.

Table 3 shows the results of the laboratory results. Compared to those without vascular calcification, patients with presence of vascular calcification had higher glycated hemoglobin and creatinine levels, while there were no differences between groups in all other variables.

Table 4 shows the results of the nutritional assessment. Compared to those without vascular calcification, patients with presence of vascular calcification had lower phase angle, lower grip strength in both arms, higher Malnutrition–Inflammation Score, and higher presence of sarcopenia and PEW. There were no differences between groups in all other variables.

Variables with significant differences between groups (*p* < 0.05) were assessed by logistic regression analyses to estimate their association with vascular calcification. Analysis with logistic regression univariate models (Table 5) showed that vascular calcification had significant association with age, diastolic blood pressure, median blood pressure, creatinine, grip strength in both arms, phase angle, Malnutrition–Inflammation Score, glycated hemoglobin, diabetes mellitus, protein energy wasting syndrome, and sarcopenia.

In order to discern the variables that are independently associated with vascular calcification, logistic regression analyses with multivariate models were performed. The predicted (dependent) variable was the presence of vascular calcification. A baseline model with clinically relevant variables included age (years), diabetes mellitus (yes or no), and mean arterial pressure (mmHg) as predictors (independent variables). Then, other multivariate models included the baseline model and one of the following variables: phase angle (degrees), Malnutrition–Inflammation Score (points), protein-energy waste syndrome (yes or no), sarcopenia (yes or no), dialysis volume (L), and serum creatinine (mg/dL). Among all tested variables, only age had an independent association with vascular calcification (Table 6). To assess the independent association of the Charlston index with vascular calcification, a model with age, mean arterial blood pressure, and the Charlston index (instead of diabetes mellitus) was tested. Only the age remained independently associated with vascular calcification (data not shown).

## 4. Discussion

We found an association between nutritional status and vascular calcification in patients undergoing automated peritoneal dialysis. The assessment of vascular calcification status is feasible in many settings and identifying patients with vascular calcification may be relevant for a better clinical follow-up [6], particularly among those with concomitant sarcopenia and PEW. Aging was the only independent factor associated with vascular calcification. That is an interesting finding considering that our populations’ age was 39 ± 14 years, which is younger than reported in previous studies [5]. This finding supports the fact of accelerated vascular aging in patients with CKD, which has been associated with increased cardiovascular mortality. 

In non-CKD, healthy subjects, aging itself it is linked to vascular damage. The specific mechanisms are characterized by changes in the expression patterns of microRNA, autophagy, migration, and proliferation of smooth muscle cells and arterial calcification [14]. Such changes produce rigidity after the second decade of life without a predilection for the size of the vessels as observed in the results of our study (large, medium, and small arteries). The decrease in elasticity and loss of the Windkessel effect (vascular recoil during systole that serves as a reservoir to attenuate the decrease in blood pressure during diastole) [15]. Histologically, this presents with degradation of elastic fibers, change in collagen content, and remodeling of the extracellular matrix, which promotes increased pulse wave velocity, increased systolic blood pressure and pulse pressure, left ventricular workload, and perfusion, finally leading to organ damage [15]. This synthetic phenotype of muscle cells and the adaptive response to the forces that act on the arterial wall cause a thickening of the intima, later this is the substrate for the formation of atherosclerotic lesions [14]. In these plaques, there is an accumulation of lipids and collagen, and in elderly patients, the inflammation decreases leading to calcification more frequently compared to that in younger patients [14]. Oxidative stress and inflammation are related to endothelial dysfunction in aging, even in the absence of clinical disease [16]. It has been reported that, compared to the general population, patients with CKD have a much-accelerated aging process characterized by progressive vascular disease, uremic inflammation, muscle wasting, osteoporosis, and frailty [17]. The process of early vascular calcification in CKD patients is predominantly characterized by media vascular calcification, a cell-mediated process primarily driven by alterations in vascular smooth muscle cells [18], and the extent of vascular calcification may be used as a measure of biological vascular age. These findings help to explain the reason we found age as the only independent factor associated to vascular calcification, even after considering the Charlson comorbidity index, which was reported as an independent factor of vascular calcification in a previous study [5]. 

Another finding in our study was that sarcopenia and PEW were associated with vascular calcification. A study that reported the results of a survey of 95 patients more than 3 months after their introduction to dialysis therapy stated that 33% had been diagnosed with sarcopenia [9]; 23.5% of our patients without vascular calcification had sarcopenia. This finding supports that adequate nutritional status may help slow down the process of vascular calcification. Kanazawa et al. reported a 14.8% incidence of PEW in Japanese dialysis patients [19], our study found 29% of PEW prevalence in patients with vascular calcification. These finding supports the importance of intensive nutritional assessment in patients undergoing automated peritoneal dialysis. These associations reflect the phenomenon of “inverse epidemiology” that has been reported in previous studies [10], which associates the presence of PEW in CKD with poor cardiovascular outcomes. Moreover, our finding of sarcopenia and PEW as factors related to vascular calcification warrant the need for future prospective trials to assess the impact of nutritional interventions to prevent vascular calcification and its negative consequences in cardiovascular mortality. 

Vascular association has been associated with laboratory parameters of bone mineral density regulation in patients with CKD treated with hemodialysis [20] or peritoneal dialysis [5]. However, we found no difference in the serum levels of parathormone, calcium, phosphorous, and alkaline phosphatase between patients with or without vascular calcification. The discrepancies between our findings may be related to differences in the renal replacement therapy [20] or in the patient’s characteristics (e.g., older age [5]). Niu et al. [5] reported that, in patients undergoing peritoneal dialysis, the parathormone was an independent factor for large artery calcification and for medium and small artery calcification. Compared with their study [5], we found lower percentages of vascular calcification in large arteries (64.9% vs. 33%) and medium arteries (42.9% vs. 33%) but a larger percentage in small arteries (9.7% vs. 25%). Unfortunately, we did not have an adequate sample size to perform a reliable analysis of the factors associated with vascular calcification according to artery size. Further studies with a larger number of patients are required to gain insight on the pathophysiology of vascular calcification of patients treated with automated peritoneal dialysis, including factors such as the biomarkers of bone mineral density regulation. 

One of this study’s limitations is its transversal design. Prospective studies in patients undergoing automated peritoneal dialysis that compare the time for vascular calcification development in patients with PEW and sarcopenia may help describe whether these two factors accelerate the progression of vascular calcification in time. Moreover, our population is different from other studies that have older patients with CKD and diabetes as the cause of CKD, which is the first cause of CKD in the general population. Other interventions to slow down vascular calcification should also be addressed, such as mineral bone disease–targeted interventions and what effect kidney transplantation has in vascular calcification. Finally, the identification of vascular calcification from radiographs may be less sensitive than other imaging techniques such as computed tomography (CT)–based imaging, as has been observed in patients treated with hemodialysis [21]. Future studies should be performed to measure the accuracy of radiology to assess the amount of vascular calcification on patients treated with automated peritoneal dialysis, in comparison with other imaging techniques and histology. Nevertheless, the diagnosis of vascular calcification with plain radiography, as applied here, remains an affordable method to detect the presence or absence of vascular calcification and has a proven prognostic value for cardiovascular morbidity and mortality [5]. Moreover, using a lateral abdominal radiograph to detect the presence or absence of vascular calcification in CKD patients is still recommended by Kidney Disease Improving Global Outcomes (KDIGO), as a reasonable alternative to CT-based imaging [22].

Future research should address the impact of other measures such as physical activity in the development of vascular calcification in patients with CKD; the effect of new therapies such as sodium thiosulfate; and, in time, the effect of vascular calcification and nutrition in cardiovascular outcomes.

## 5. Conclusions

There is association between vascular calcification, PEW, and sarcopenia in patients with maintenance automated peritoneal dialysis. These associations are not independent of age. This is the first study that compares vascular calcification with nutritional status in automated peritoneal dialysis patients. Our patients are relatively young, so our findings suggest that, in time, CKD is associated with premature vascular aging. The assessment of vascular calcification with X-rays is a simple method that can be used to stratify patients with increased risk for cardiovascular and nutritional complications. Further studies should be done to address the pattern of progression of vascular calcification in CKD and the interventions that are useful to slow down or prevent this phenomenon. 

## Figures and Tables

**Table 1:** Distribution of vascular calcification in different arteries (N = 21).

Large Artery	Medium Artery	Small Artery	Patients
−	−	+	0 (0.0%)
−	+	−	3 (14.3%)
+	−	−	3 (14.3%)
−	+	+	3 (14.3%)
+	+	−	2 (9.5%)
+	−	+	1 (4.8%)
+	+	+	9 (42.9%)

**Table 2:** Demographic and clinical characteristics of all patients. Data are shown as absolute value (percentage), mean ± standard deviation, or median (percentile 25–percentile 75).

Variables	All Patients (N = 51)	Vascular Calcification		*p* Value
		Yes (N = 21)	No (N = 30)	
Age (years)	39 ± 14	49 ± 13	33 ± 11	<0.001
Gender				0.227
Female	24 (47)	12 (57)	12 (40)	
Male	27 (53)	9 (43)	18 (60)	
Time from CKD diagnosis (months)	60 (26–119)	82 (44–192)	51 (24–102)	0.119
Time in renal replacement therapy (months)	40 (17–84)	49 (10–103)	39 (20–76)	0.752
Time in automated peritoneal dialysis (months)	25 (6–48)	30 (6–68)	22 (8–38)	0.270
Dialysis volume (L)	10.3 ± 1.78	9.7 ± 1.7	10.8 ± 1.7	0.030
Dialysis treatment duration (hours)	10 (9–10)	9.5 (9–10)	10 (9–10)	0.470
Dialysis prescription including final infusion	27 (53)	8 (38)	19 (63)	0.076
Primary renal disease				0.220
Not determined	28 (55)	9 (43)	19 (63)	
Diabetic nephropathy	14 (28)	9 (43)	5 (17)	
Lupus nephritis	3 (6)	1 (5)	2 (7)	
Other	6 (12)	2 (9)	4 (13)	
Diabetes	14 (27.5)	10 (48)	4 (13)	0.007
Hypertension	33 (64.7)	13 (62)	20 (67)	0.726
Hyperparathyroidism	12 (32)	3 (23)	9 (36)	0.333
Prior kidney transplant	12 (23.5)	6 (29)	6 (20)	0.478
Charlson comorbidity index	2 (2–4)	3 (2–5)	2 (2–2)	0.007
Body mass index (kg/m^2^)	24.11 ± 4.21	24 ± 3.68	24 ± 4.61	0.634
Systolic blood pressure (mmHg)	143 ± 24	136 ± 28	148 ± 20	0.261
Diastolic blood pressure (mmHg)	84 ± 17	78 ± 14	89 ± 17	0.023
Mean blood pressure (mmHg)	103 ± 17	97 ± 16	108 ± 16	0.026
Pulse pressure (mmHg)	60 ± 19	61 ± 23	60 ± 17	0.922
Urinary output (mL)	0 (0–800)	160 (0–800)	0 (0–700)	0.294
Residual renal function (mL/min)	0 (0–2.82)	0.04 (0–3)	0 (0–0.36)	0.137

**Table 3:** Laboratory results. Data are shown as mean ± standard deviation or median (percentile 25–percentile 75).

Variables	All Patients (N = 51)	Vascular Calcification		*p* Value
		Yes (N = 21)	No (N = 30)	
Hemoglobin (g/dL)	10.11 ± 1.18	9.9 ± 2.7	10.3 ± 2.7	0.697
Glucose (mg/dL)	104 ± 50	106.1 ± 41.4	102.8 ± 55.9	0.981
Glycated hemoglobin (%)	5.3 (5.0–5.8)	5.56 (5.1–7.2)	5.21 (4.96–5.4)	0.014
Total cholesterol (mg/dL)	186 ± 50	192 ± 53	181 ± 48	0.891
High-density lipids (mg/dL)	41.2 ± 10.7	40.1 ± 9.8	42 ± 8.9	0.502
Low-density lipids (mg/dL)	98.6 ± 33.6	97.7 ± 35.3	99.2 ± 33	0.961
Triglycerides (mg/dL)	186 ± 123	213 ± 51	167 ± 97	0.175
Uric acid (mg/dL)	6.57 ± 1.37	6.8 ± 1.43	6.41 ± 1.34	0.808
Albumin (g/dL)	3.69 ± 0.49	3.55 ± 0.45	3.79 ± 0.5	0.251
Parathormone (pg/mL)	388 (148–614)	395 (165–644)	375 (134–501)	0.363
Corrected calcium (mg/dL)	9.07 ± 1.11	9.15 ± 0.76	9.24 ± 1.18	0.698
Phosphorus (mg/dL)	5.85 ± 1.34	5.73 ± 1.52	5.93 ± 1.21	0.571
Alkaline phosphatase (IU/L)	106.5 (85–161)	106 (93–138)	104 (83–166)	0.752
C reactive protein (mg/L)	1.65 (0.75–4.01)	2.02 (1.3–4)	1.52 (0.73–3.5)	0.363
Ferritin (ng/mL)	166.5 (79–335)	157 (62–369)	167 (86–319)	0.738
Creatinine, mg/dL	13.02 ± 4.14	11.31 ± 4.02	14.22 ± 3.85	0.012
Blood urea nitrogen (mg/dL)	55.71 ± 11.51	55.81 ± 11.98	55.6 ± 11.3	0.811
Hemoglobin (g/dL)	10.11 ± 1.18	9.9 ± 2.7	10.3 ± 2.7	0.697

**Table 4:** Nutritional assessment of 51 patients with automated peritoneal dialysis. Data are shown as absolute value (percentage), mean ± standard deviation, or median (percentile 25–percentile 75).

Variables	All Patients (N = 51)	Vascular Calcification		*p* Value
		Yes (N = 21)	No (N = 30)	
Weight (kg)	62.5 ± 12.5	60 ± 12.8	63.7 ± 12.3	0.311
Height (m)	1.60 ± 0.09	1.58 ± 0.10	1.62 ± 0.08	0.076
Body mass index (kg/m^2^)	24.1 ± 4.2	23.9 ± 3.7	24.2 ± 4.6	0.806
Overhydration (kg)	0 (0–0.7)	0 (0–0.8)	0 (0–0.5)	0.303
Phase angle (degrees)	4.77 ± 0.85	4.32 ± 0.82	5.09 ± 0.73	0.001
Fat-free mass (kg)	42.9 ± 9.7	40.4 ± 7.9	44.6 ± 10.5	0.131
Fat free mass index (kg/m^2^)	16.6 ± 3	16.18 ± 2.25	16.89 ± 3.44	0.409
Right arm hand grip strength (kg)	25 ± 12	18 ± 9	29 ± 12	0.001
Left arm hand grip strength (kg)	23 ± 12	17 ± 9	28 ± 12	0.001
Malnutrition–Inflammation Score	5 ± 3	6 ± 4	4 ± 2	0.011
Sarcopenia	12 (23.5)	8 (38)	4 (13)	0.040
Protein-energy wasting syndrome	8 (15.7)	8 (15.7)	2 (7.0%)	0.034

**Table 5:** Logistic regression analysis with univariate models and vascular calcification as dependent variable.

Independent Variables	Odds Ratio (95% CI)	*p* Value
Age (years)	1.105 (1.044–1.169)	0.001
Diabetes mellitus	5.909 (1.52–22.95)	0.010
Diastolic blood pressure (mmHg)	0.957 (0.919–0.996)	0.031
Mean blood pressure (mmHg)	0.96 (0.925–0.997)	0.033
Glycated hemoglobin (%)	2.48 (1.182–5.222)	0.016
Creatinine (mg/dL)	0.827 (0.706–0.967)	0.018
Grip strength, left arm (kg)	0.903 (0.844–0.966)	0.003
Grip strength, right arm (kg)	0.895 (0.83–0.965)	0.004
Phase angle (degrees)	0.279 (0.119–0.653)	0.003
Malnutrition–Inflammation Score	1.32 (1.041–1.692)	0.022
Protein-energy wasting syndrome	5.6 (1.004–31.2)	0.049
Sarcopenia	4 (1.014–15.78)	0.048

**Table 6:** Logistic regression analysis with multivariate models and vascular calcification as dependent variable.

Independent Variables	Odds Ratio (95% CI)	*p* Value
Age (years) *	1.092 (1.025–1.164)	0.006
Diabetes mellitus *	2.385 (0.416–13.66)	0.329
Mean arterial pressure (mmHg) *	0.96 (0.910–1.014)	0.143
Phase angle ^&^	0.333 (0.101–1.099)	0.071
Malnutrition–Inflammation Score ^&^	1.184 (0.849–1.651)	0.329
Protein-energy wasting syndrome ^&^	3.969 (0.363–43.372)	0.258
Sarcopenia ^&^	0.593 (0.104–3.38)	0.557
Dialysis volume ^&^	0.688 (0.426–1.11)	0.126
Serum creatinine ^&^	0.922 (0.739–1.15)	0.471

* Variable included in the baseline model. ^&^ Variable added to the baseline model.

## Data Availability

The data presented in this study are available on request from the corresponding author. The data are not publicly available to protect the study participants from potential identification based on their personal information provided in the study variables.
